# Harmonic Distortion Optimization for Sigma-Delta Modulators Interface Circuit of TMR Sensors

**DOI:** 10.3390/s20041041

**Published:** 2020-02-14

**Authors:** Xiangyu Li, Jianping Hu, Xiaowei Liu

**Affiliations:** 1 Faculty of Information Science and Engineering, Ningbo University, Ningbo 315211, China; lixiangyu@nbu.edu.cn; 2 MEMS Center, Harbin Institute of Technology, Harbin 150001, China; xiaoweiliu.hit@gmail.com

**Keywords:** harmonic distortion, interface circuit, tunneling magneto-resistance sensors

## Abstract

The tunneling magnetoresistance micro-sensors (TMR) developed by magnetic multilayer material has many advantages, such as high sensitivity, high frequency response, and good reliability. It is widely used in military and civil fields. This work presents a high-performance interface circuit for TMR sensors. Because of the nonlinearity of signal conversion between sensitive structure and interface circuit in feedback loop and forward path, large harmonic distortion occurs in output signal spectrum, which greatly leads to the reduction of SNDR (signal noise distortion rate). In this paper, we analyzed the main source of harmonic distortion in closed-loop detection circuit and establish an accurate harmonic distortion model in TMR micro-sensors system. Some factors are considered, including non-linear gain of operational amplifier unit, effective gain bandwidth, conversion speed, nonlinearity of analog transmission gate, and nonlinearity of polycrystalline capacitance in high-order sigma-delta system. We optimized the CMOS switch and first-stage integrator in the switched-capacitor circuit. The harmonic distortion parameter is optimally designed in the TMR sensors system, aiming at the mismatch of misalignment of front-end system, non-linearity of quantizer, non-linearity of capacitor, and non-linearity of analog switch. The digital output is attained by the interface circuit based on a low-noise front-end interface circuit and a third-order sigma-delta modulator. The digital interface circuit is implemented by 0.35μm CMOS (complementary metal oxide semiconductor) technology. The high-performance digital TMR sensors system is implemented by double chip integration and the active interface circuit area is about 3.2 × 2 mm. The TMR sensors system consumes 20 mW at a single 5 V supply voltage. The TMR sensors system can achieve a linearity of 0.3% at full scale range (±10^5^ nT) and a resolution of 0.25 nT/Hz^1/2^(@1Hz).

## 1. Introduction

Recently, high-precision tunneling magneto-resistance sensors (TMR) can combined with inertial sensors are widely used in GPS-aided navigators for the consumer market, geomagnetic signal measurements in space [1,2]. TMR sensors have the advantages of low power consumption, miniaturization, good stability, and easy integration with CMOS process [3,4,5]. So high-performance TMR sensors with an accuracy of sub-nT level occupy a large market share in inertial navigation, space microgravity measurement, platform stability control, and other fields. It is particularly important to study the interface circuit of high-performance TMR sensors. The high-performance TMR sensors have strict requirements on the output signal distortion, which usually use a sigma-delta modulator to form closed-loop control system. However, the analysis and optimization of a TMR sensor system’s harmonic distortion parameters need further research.

In this paper, we analyzed the main source of harmonic distortion in closed-loop detection circuit and proposed a third-order sigma-delta interface circuit. The interface circuit can provide direct digital output and avoid the use of high-precision ADCs for the analogue front-end, which eliminates the deterioration of overall noise floor. Switched-capacitor (S-C) interface circuit for sigma-delta modulators can be obtained in CMOS process easily. Harmonic distortion and noise are the key parameters which determine the performance of TMR sensors. The tunneling magneto-resistance micro-magnetometers can achieve a high-precision output (less than 1 nT/Hz^1/2^) by input-stage chopping and ripple suppression loop [6,7]. We proposed some optimization methods of harmonic distortion for high-precision miniaturized three-channel TMR sensors interface circuit. The analog front-end interface ASIC (application specific integrated circuit) chip for tunneling magneto-resistive sensors are implanted by 0.35 μm CMOS technology. The test results show that: a power consumption of 20mW, a resolution of 0.25 nT/Hz^1/2^(@1 Hz), a linearity of lower than 0.1% full scale and a chip area of 3.2 × 2 mm^2^.

The TMR sensitive element and interface circuit are introduced and designed in Section 2. In Section 3, we show the detailed measurement results of ASIC interface circuit and TMR sensors system. Finally, Section 4 concludes this work.

## 2. Materials and Methods

### 2.1. Materials

The TMR sensitive element with multilaminar structure is from Multidimension Technologies (Suzhou, China). The interface circuits based on TMR sensors are implemented by 0.35 μm CMOS process and cooperated with Shanghai Huahong Integrated Circuit Co., Ltd (Shanghai, China).

### 2.2. TMR Sensitive Element and Interface Circuit

**Figure 1:**
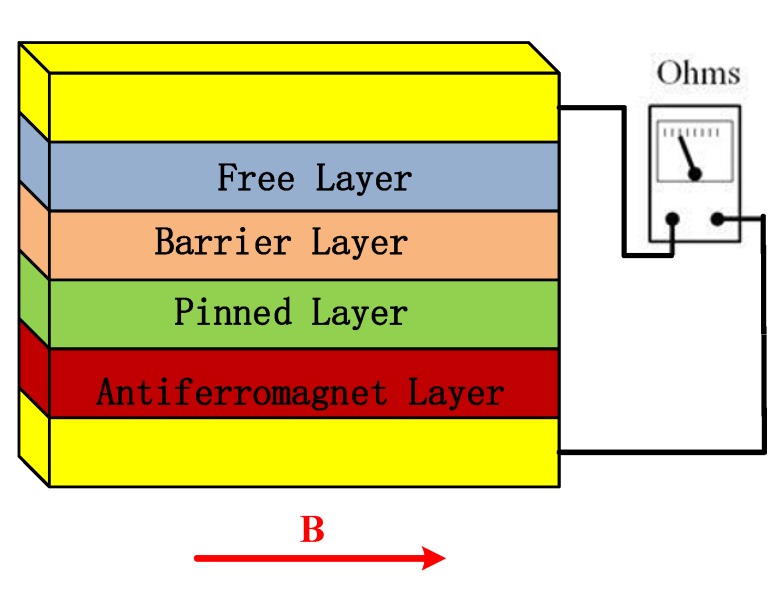
Tunneling magnetic resistance-type sensitive structure.

The miniaturized solid-state magnetometers mainly include Hall-effect magnetometers, anisotropic magneto-resistance, giant magneto-resistance, and tunneling magneto-resistance [8,9]. The TMR element with multilayer film structure has created more and more applications in the magnetometer devices due to its high sensitivity and low-power consumption [10]. The sensitive structure part of tunneling magneto-resistive sensor mainly consists of pinning layer, tunnel barrier, and free layer. The pinning layer composed of ferromagnetic layer and anti-ferromagnetic layer (AFM layer). The exchange coupling between ferromagnetic layer and anti-ferromagnetic layer determines the direction of the magnetic moment of a ferromagnetic layer; tunneling barrier layer is usually composed of MgO or Al_2_O_3_, located in the upper part of anti-ferromagnetic layer [11]. As shown in Figure 1 the arrows represent the direction of the magnetic moment of the pinning layer and the free layer. The magnetic moment of the pinning layer is relatively fixed under the action of a certain size of magnetic field. The magnetic moment of the free layer is relatively free and rotatable to the magnetic moment of the pinning layer, and it will turn over with the change of the magnetic field. The typical thickness of each film layer is between 0.1 nm and 10 nm [12,13,14]. The sensitive element concludes 32 magnetic tunneling junctions (MTJ). The area of magnetic tunneling junctions is 50 μm^2^. In this work, the thickness of free layer/barrier layer/pinning layer is 10/1/10 nm. The multilayer structure of MTJ is Ta/Ru/Ta/PtMn/CoFe/Ru/CoFeB/MgO/CoFeB/NiFe/Ru/Ta. Thin film is deposited by magnetron sputtering. MgO materials are used in the barrier layer so that TMR element is more sensitive and higher resolution. A Wheatstone bridge configuration composed of four active TMR arrays are applied by thin film process. The three-axis TMR sensitive element is built by stereoscopic orthogonal package. The sensitive element with flux modulation structure used for design, simulation and test in this work is from the Multidimension Technology Company. The sensitive element can achieve a background noise of 150 pT/Hz^1/2^ by the vertical modulation film and a power consumption of 12.5 mW at 5 V power supply. Major parameter indicators are shown as in Table 1. 

**Figure 2:**
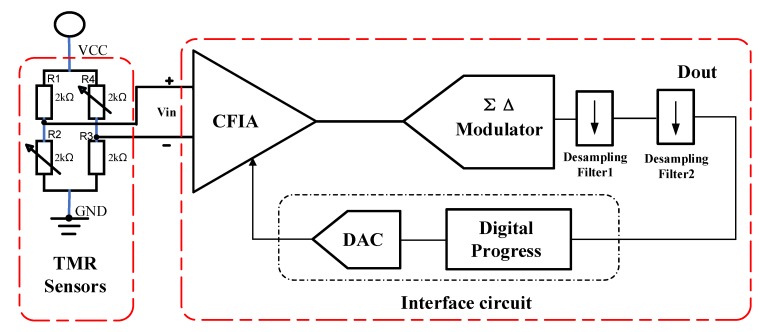
Interface circuit based on TMR sensors.

**Figure 3:**
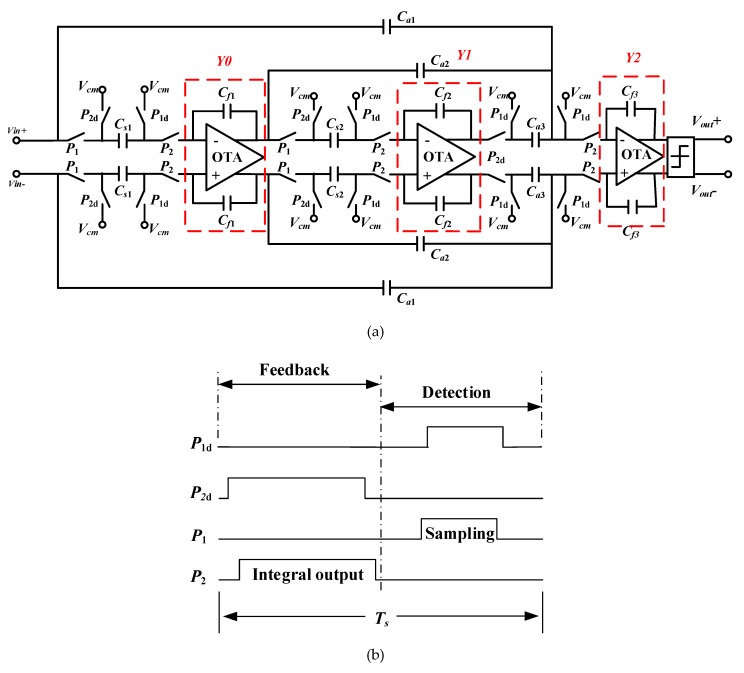
Third-order CIFF sigma-delta modulator circuit and the working sequence: (**a**) sigma-delta modulator circuit; (**b**) working sequence diagram.

The read-out interface circuit of TMR sensors is consisted of a current feedback instrumentation amplifier circuit (CFIA), a sigma-delta modulator and desampling filters as shown in Figure 2. For a tunneling magneto-resistance sensor element, a current feedback instrumentation amplifier circuit is used for the preceding stage weak signal detection. The main noise source of the system comes from low-frequency 1/*f* noise. In order to eliminate low-frequency noise of sensors and improve the SNR of bandgap reference, the chopper stabilization technique is applied. The analog signals are converted into high-precision digital signals by sigma-delta ADC. We proposed the third-order CIFF (cascade-of-integrators feed-forward) sigma-delta interface circuit and the working sequence as shown in Figure 3. The first stage switched capacitor integrator is the key unit of sigma-delta modulator system to realize loop filtering. Because the discrete signals are processed in switched capacitor circuit, the nonlinear analysis of the switched capacitor integrator is mainly in the discrete time domain. The timing diagram of the sigma-delta is as shown in Figure 3b. There are four phases in operation of the circuit which is feedback phase, detection phase, sampling phase, and integral output phase. Wherein P1 and P2 are the two-phase non-overlapping clock, P1 is active-high, P2 is active-low. The shutdown time of P1d is later than P1, the shutdown time of P2d is later than P2, it can effectively suppress the influence of charge injection and clock-feedthrough in the switched-capacitor circuit. The feedback and detection phase operate at different times of a cycle to eliminate noise coupling. In the sampling phase, the input voltage signal is reset to ensure a correct bias point and the sampling capacitor is discharged to erase the memory from the previous cycle. The nonlinearity of switched capacitor integrators mainly originates from non-ideal factors of operational amplifier, such as non-linear DC gain, limited gain bandwidth, and limited voltage swing rate of op-amp which can lead to non-linearity during the transient establishment of integrators and generating high-order harmonic distortion in the system output. Considering the influence of the finite non-linear DC gain of the operational amplifier on the integrator nonlinearity, the DC gain of the operational amplifier is finite and varies with the output voltage [15]. This can lead to harmonic distortion of the sigma-delta system.

### 2.3. Analysis and Optimization of Harmonic Distortion

**Figure 4:**
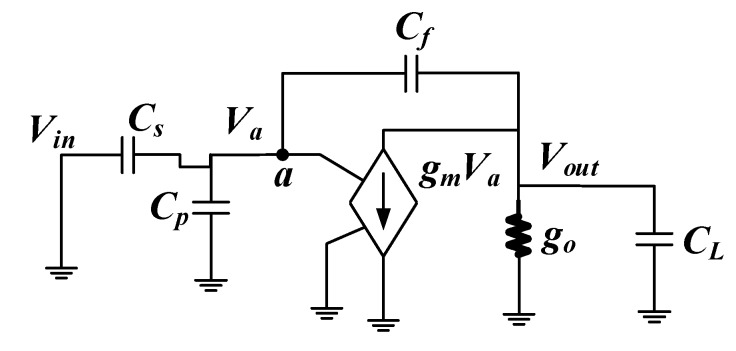
Integrator model in the integration-stage.

The non-ideal factors of operational amplifier mainly lead to the non-linearity of integrator in the integration stage. The equivalent non-ideal model of integrator is as shown in Figure 4 in the integration stage. *C_s_, C_f_, C_p_*, and *C_L_* are sampling capacitors, integral feedback capacitors, parasitic capacitors, and load capacitors, *A* is operational amplifier gain, *V_in_* and *V_o_* are input and output signal respectively, *V_a_* is the potential at *a* point, *g_m_* and *g_o_* are the input and output transconductance of operational amplifier respectively.

According to the input-output relationship of the operational amplifier, where gain *A* varies with the output voltage

(1) $$A(V_{o})=A_{o}(1+a_{1}V_{o}+a_{2}V_{o}^{2}+a_{3}V_{o}^{3}\cdots )$$

For a fully differential structure, if $A(V_{o})$ is an even function, its odd coefficients are all zero.

(2) $$A(V_{o})=A_{o}(1+a_{2}V_{o}^{2}+a_{4}V_{o}^{4}\cdots )$$

Among them, the parameters *a_2_* and *a_4_* can be determined by the gain non-linear model [16].

(3) $$\left\{ \begin{array}{l} a_{2}=-9{\left[\frac{A_{0}^{0.01}}{{\left(1+V_{os}\right)}^{2.6}}\right]}^{2}\\ a_{4}=-6{\left[\frac{A_{0}^{0.01}}{{\left(1+V_{os}\right)}^{0.83}}\right]}^{4} \end{array}\right.$$

In the mode, *A_0_* is the DC gain of the operational amplifier and *V_os_* is the output voltage swing.

According to integration stage model in the integrator, in the initial state, assuming that *C_L_* value is very large, it can be obtained from the charge conservation.

(4) $$\left\{ \begin{array}{l} V_{a}(0^{+})\approx -\frac{K_{s}}{K_{p}+K_{s}+1}V_{in}(nT_{s}-T_{s})-\frac{K_{p}+1}{A(K_{p}+K_{s}+1)}V_{o}(nT_{s}-T_{s})\\ V_{o}(nT_{s}-\frac{T_{s}}{2}+0^{+})=V_{n}(0^{+})=V_{o}(nT_{s}-T_{s})+\frac{C_{L}}{C_{L}+C_{f}}[V_{a}(0^{+})+\frac{1}{A}V_{o}(nT_{s}-T_{s})]\approx V_{o}(nT_{s}-T_{s}) \end{array}\right.$$

Among them, $K_{s}=\frac{C_{s}}{C_{f}}$, $K_{p}=\frac{C_{p}}{C_{f}}$, *T_s_* is the sampling clock cycle. In the integral stage, the transient current equation is

(5) $$\left\{ \begin{array}{l} \left(C_{p}+C_{s})\frac{dV_{a}(t)}{dt}=C_{f}\frac{d}{dt}[V_{n}(t)-V_{a}(t)\right]\\ C_{f}\frac{d}{dt}[V_{n}(t)-V_{a}(t)]+C_{L}\frac{dV_{n}(t)}{dt}+g_{o}V_{n}(t)=-I(t) \end{array}\right.$$

(6) $$I(t)=\left\{ \begin{array}{l} g_{m}V_{a}(t),|V_{a}(t)|\leq I_{o}/g_{m}\\ I_{o},|V_{a}(t)|>I_{o}/g_{m} \end{array}\right.$$

According to the above results, the integral establishment is analyzed:

①If $|V_{a}(0^{+})|\leq I_{o}/g_{m}$, $K_{s}|V_{in}(nT_{s}-T_{s})|\leq \frac{I_{o}}{C_{eff}}\cdot \frac{C_{eff}}{g_{m}}(K_{s}+K_{p}+1)$, this is the transient establishment process of integral stage.

Among them, $\beta =\frac{g_{m}}{C_{eff}}(1+\frac{K_{s}+K_{p}+1}{A})$, $C_{eff}=C_{s}+C_{p}+C_{L}(K_{s}+K_{p}+1)$, $A=\frac{g_{m}}{g_{o}}\gg 1$, operational amplifier voltage pendulum rate(*SR*) can be expressed as: $SR=\frac{I_{o}}{C_{eff}}$, unit gain bandwidth product can be expressed as: $GBW=\frac{1}{2\pi }\cdot \frac{g_{m}}{C_{eff}}$ , time constant can be expressed as $\tau =\frac{1}{2\pi GBW}=\frac{C_{eff}}{g_{m}}$ .

We can obtain *V_n_(t)* at transient establishment phase of integrator

(7) $$\frac{K_{s}|V_{in}(nT_{s}-T_{s})|}{(K_{s}+K_{p}+1)\tau }\leq SR$$

(8) $$\begin{array}{l} V_{n}(t)=K_{s}\frac{A}{A+K_{s}+K_{p}+1}V_{in}(nT_{s}-T_{s})(1-e^{-\beta t})\\ +(\frac{A+K_{p}+1}{A+K_{s}+K_{p}+1}+\frac{K_{s}}{A+K_{s}+K_{p}+1}e^{-\beta t})V_{o}(nT_{s}-T_{s}) \end{array}$$

Among them, $g=\frac{A}{A+K_{s}+K_{p}+1}$, $a=\frac{A+K_{p}+1}{A+K_{s}+K_{p}+1}$, $\beta =\frac{1}{g\tau }$. At the end of the integral, *t = T_s_/2*, the output of the sigma-delta system can be expressed as

(9) $$V_{o}(nT_{s})=V_{n}(\frac{T_{s}}{2})=K_{s}gV_{in}(nT_{s}-T_{s})(1-e^{-\frac{T_{s}}{2g\tau }})+[a+(1-a)e^{-\frac{T_{s}}{2g\tau }}]V_{o}(nT_{s}-T_{s})$$

The results of the equation show that when the swing rate is large enough, if the nonlinearity of DC gain is neglected, there is no nonlinearity in the integrator output, which indicates that the limited swing rate and bandwidth of the operational amplifier will not lead to nonlinearity at the integrator establishment process. According to the generation mechanism of harmonic distortion in discrete time domain, the nonlinearity of the integrator is only caused by the nonlinear gain of operational amplifier.

②When $|V_{a}(0^{+})|>I_{o}/g_{m}$, $\frac{K_{s}|V_{in}(nT_{s}-T_{s})|}{(K_{s}+K_{p}+1)\tau }>SR$, limited swing rate and bandwidth of operational amplifier may lead to the nonlinearity of integrator transient establishment

(10) $$t\leq t_{0}=\frac{C_{eff}}{I_{o}}[\frac{K_{s}|V_{in}(nT_{s}-T_{s})|}{K_{s}+K_{p}+1}-\frac{I_{o}}{g_{m}}]=\frac{K_{s}|V_{in}(nT_{s}-T_{s})|}{(K_{s}+K_{p}+1)SR}-\tau$$

The equation at the transient establishment process of integral stage can be expressed as

(11) $$\left\{ \begin{array}{l} V_{a}(t)\approx V_{a}(0^{+})-\frac{I_{o}}{C_{eff}}t\mathrm{sgn}[V_{a}(0^{+})]\\ V_{n}(t)=(K_{s}+K_{p}+1)[V_{a}(t)-V_{a}(0^{+})]+V_{n}(0^{+}) \end{array}\right.$$

When $\frac{T_{s}}{2}\leq t_{0}$, at the end of the integral, the output of the sigma-delta system can be expressed as

(12) $$V_{o}(nT_{s})=V_{n}(\frac{T_{s}}{2})\approx V_{o}(nT_{s}-T_{s})+(K_{s}+K_{p}+1)\frac{T_{s}}{2}SR\mathrm{sgn}[V_{i}(nT_{s}-T_{s})]$$

The final output of the sigma-delta system can be expressed as

(13) $$\begin{array}{l} V_{o}(nT_{s})=V_{n}(\frac{T_{s}}{2})=K_{s}gV_{in}(nT_{s}-T_{s})+[a+(1-a)e^{-\frac{T_{s}}{2g\tau }+\frac{t_{0}}{g\tau }}]V_{o}(nT_{s}-T_{s})\\ -\mathrm{sgn}[V_{i}(nT_{s}-T_{s})](K_{s}+K_{p}+1)SR\tau e^{-\frac{T_{s}}{2g\tau }+\frac{t_{0}}{g\tau }} \end{array}$$

**Figure 5:**
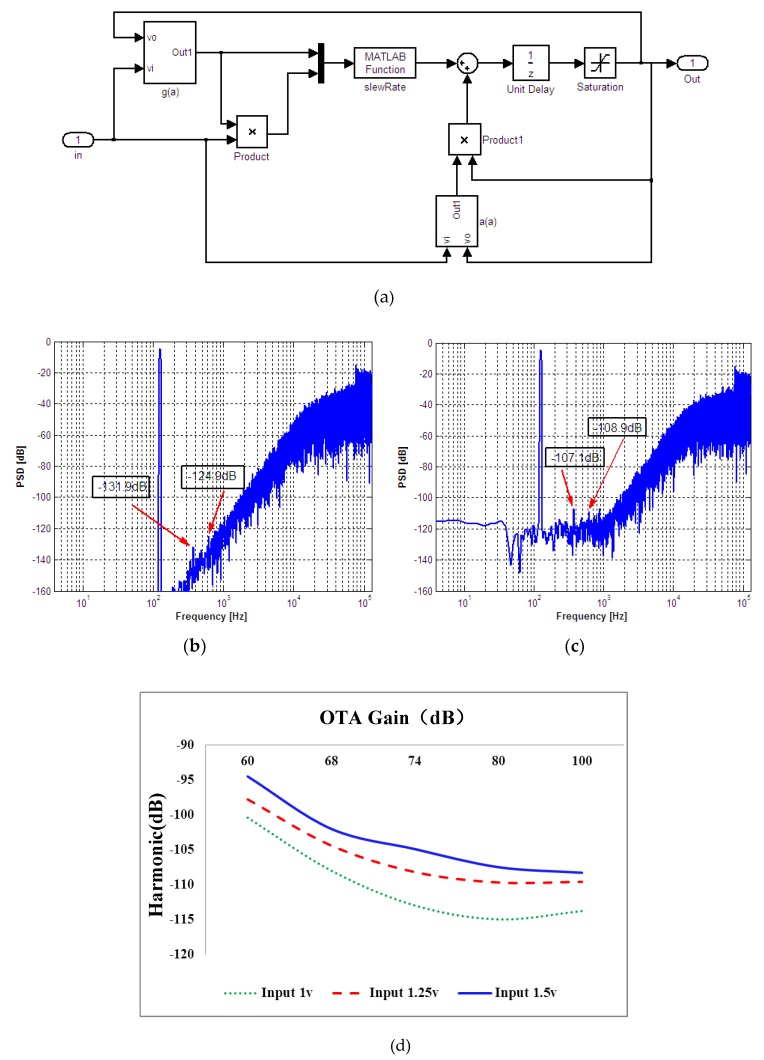
Integrator nonlinearity model and simulation: (**a**) Integrator nonlinearity model in Simulink; (**b**) PSD simulation of ideal model; (**c**) PSD simulation of Integrator nonlinearity model (**d**) Harmonic distortion changes with operational amplifier gain.

In the Equation (13) *t_0_* is related to the input signal. Even if the nonlinearity of operational amplifier gain is neglected, the nonlinearity of integrator output can lead to system output harmonics. We summarize the above analysis results: for the given swing rate and bandwidth of operational amplifier, when the input signal amplitude is small, the final output of integrator is given by Equation (9). There is no nonlinearity in the integrator. When the amplitude of input signal increases to a certain value, the integrator output is determined by Equation (13). Obviously, the establishment process of integrator is non-linear at this time. According to Equation (9), Equation (12), Equation (13), gain nonlinearity in the Equation (2) and Equation (3), the nonlinear model of integrator can be established as shown in Figure 5a.

In order to verify the analysis results and the established model, we add the model as shown in Figure 5a to the ideal third-order electrical modulator model and then simulate. The dynamic simulation of the modulator is carried out by changing the DC gain of the operational amplifier, and the output results are analyzed. Because the typically output from TMR element is ac signal at the millivolt range. In simulation, we set the input sine wave signal as a frequency of 125 Hz, an amplitude of 1V. The PSD (power spectral density) output of the ideal model is compared with that of the model with nonlinear integrator as shown Figure 5b,c. In the integrator, the DC gain of the operational amplifier gain is 68 dB, the voltage swing rate is 40 mV/s, and the unit gain bandwidth product is 40 MHz. It can be seen from the figure that the harmonic distortion of the system increases obviously after the integrator nonlinearity is added. In order to further analyze the influence of operational amplifier gain, we set a signal frequency of 250 Hz as the input signal and change the operational amplifier gain and input signal amplitude. The third harmonic distortion of the system changing with operational amplifier gain is shown in Figure 5d. Due to the influence of operational amplifier nonlinear gain, as the operational amplifier gain decreases, the output harmonic distortion of the system will increase.

The switch is a key module in the switched-capacitor (S-C) sigma-delta modulator circuit. The nonlinearity will have a great influence on the linearity of the system [17]. The nonlinearity of the switch mainly includes on resistance nonlinearity and channel charge injection nonlinearity [18]. If only NMOS or PMOS is used as switch, the *R_on_* (conduction resistance) will change nonlinearly with the input signal, this will introduce harmonic distortion to the system. The CMOS complementary switch is commonly used in switched-capacitor circuit. We set the coefficient *K_N_* and *K_P_* as the Equation (14).

(14) $$\left\{ \begin{array}{l} K_{N}=\mu_{N}C_{ox}\frac{W_{N}}{L_{N}}\\ K_{P}=\mu_{P}C_{ox}\frac{W_{P}}{L_{P}} \end{array}\right.$$

The *R_on_* (conduction resistance) of the switch can be expressed as

(15) $$\begin{array}{l} R_{on}^{-1}=R_{on,N}^{-1}+R_{on,P}^{-1}=K_{N}(V_{DD}-V_{in}-V_{THN})+K_{P}(V_{in}+V_{THP}-V_{SS})\\ =(K_{N}V_{DD}-K_{P}V_{SS})-(K_{N}-K_{P})V_{in}-(K_{N}V_{THN}-K_{P}V_{THP}) \end{array}$$

If we ignore the substrate bias effect, then design the suitable size $K_{N}=K_{P}$. The linearity of the switch will be optimized. If we consider the substrate bias effect, the threshold voltage *V_THN_* and *V_THP_* can be expressed as

(16) $$\left\{ \begin{array}{l} V_{THN}=V_{THN0}+\gamma_{N}(\sqrt{|2\phi_{F}|+V_{in}-V_{SS}}-\sqrt{\left|2\phi_{F}\right|})\\ V_{THP}=V_{THP0}-\gamma_{P}(\sqrt{|2\phi_{F}|+V_{DD}-V_{in}}-\sqrt{\left|2\phi_{F}\right|}) \end{array}\right.$$

So we can obain the Equation (17), in the Equation (17) *V_1_* and *V_2_* can be expressed as

(17) $$R_{{}_{on}}^{-1}\approx KV_{1}-K(\gamma_{N}\sqrt{V_{2}}+\gamma_{P}\sqrt{V_{2}})-\frac{1}{2}\frac{K}{\sqrt{V_{2}}}(\gamma_{N}-\gamma_{P})V_{in}+\frac{1}{8}\frac{K}{{\sqrt{V_{2}}}^{3}}(\gamma_{N}+\gamma_{P})V_{in}{}^{2}$$

(18) $$\left\{ \begin{array}{l} V_{1}=V_{DD}-V_{SS}-V_{THN0}+V_{THP0}+\gamma_{N}\sqrt{\left|2\phi_{F}\right|}+\gamma_{P}\sqrt{\left|2\phi_{F}\right|}\\ V_{2}=|2\phi_{F}|+V_{DD} \end{array}\right.$$

In general,$\gamma_{N}\approx \gamma_{P}=\gamma$, the Equation (17) can be simplified as

(19) $$R_{on}^{-1}\approx KV_{1}-2K\gamma \sqrt{V_{2}}+\frac{1}{4}\frac{K}{{\sqrt{V_{2}}}^{3}}\gamma V_{in}^{2}$$

Due to the substrate bias effect, the conduction resistance of CMOS complementary switch still has some nonlinearity. In addition, the conduction resistance of the switch will also affect the integrator. In the sampling phase of integrator, the conduction resistance of switches *P_1_* and *P_1d_* can be expressed as

(20) $$R_{{}_{on}}^{-1}=R_{{}_{on,N}}^{-1}+R_{{}_{on,P}}^{-1}=K_{N}(V_{DD}-V_{in}-V_{THN})+K_{P}(V_{in}+V_{THP}-V_{SS})$$

At the end of sampling, the amount of charge on the capacitance C_S_ can be expressed as

(21) $$Q=C_{s}V_{in}(nT_{s}-T_{s})(1-\varepsilon_{s})$$

In the Equation (21), $\varepsilon_{s}=e^{-T_{s}/(4R_{on}C_{s})}$. In the integration stage, the actual amount of charge transfer stored on C_S_ can be expressed as

(22) $$Q’=C_{s}V_{in}(nT_{s}-T_{s})(1-\varepsilon_{s})(1-\varepsilon_{i})$$

In the Equation (22), $\varepsilon_{i}\approx \varepsilon_{s}$. The signal transfer function and transfer function of integrator can be expressed as

(23) $$V_{o}(nT_{s})-V_{o}(nT_{s}-T_{s})=\frac{C_{s}}{C_{f}}{\left(1-\varepsilon_{s}\right)}^{2}V_{in}(nT_{s}-T_{s})$$

(24) $$H(z)=\frac{C_{s}}{C_{f}}\frac{z^{-1}}{1-z^{-1}}{\left(1-e^{-\frac{T_{s}}{4R_{on}C_{s}}}\right)}^{2}$$

**Figure 6:**
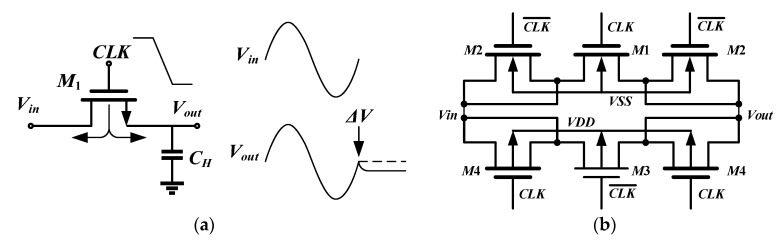
Switch nonlinearity model and six-transistor complementary switch: (**a**) The channel charge injection model; (**b**) Circuit diagram of six-transistor complementary switch.

In addition, the channel charge injection effect and clock feedthrough effect of MOS transistor are the main causes of switching nonlinearity. The channel charge injection model is shown in Figure 6a. When the switch is on, the total charge *Q_ch_* in the inversion layer can be expressed as

(25) $$Q_{ch}=WLC_{ox}(V_{DD}-V_{in}-V_{TH})$$

When the switch is off, the charge will flow out through the source end and the drain end. The ratio of charge injection to capacitance *C_H_* is related to the ratio of total capacitance, threshold voltage, input voltage and width-to-length ratio. The error voltage of the output in the CMOS complementary switch can be expressed as

(26) $$\Delta V=\frac{W_{N}L_{N}C_{ox}(V_{DD}-V_{in}-V_{THN})}{2C_{H}}-\frac{W_{P}L_{P}C_{ox}(V_{in}-|V_{THP}|-V_{SS})}{2C_{H}}$$

In the design of switch, we set: $K_{H}=\frac{W_{N}L_{N}C_{ox}}{2C_{H}}=\frac{W_{P}L_{P}C_{ox}}{2C_{H}}$ and $V_{DD}=-V_{SS}$. The output *V_o_* can be expressed as

(27) $$V_{o}=V_{in}-\Delta V=V_{in}-[\frac{W_{N}L_{N}C_{ox}(V_{DD}-V_{in}-V_{THN})}{2C_{H}}-\frac{W_{P}L_{P}C_{ox}(V_{in}-|V_{THP}|-V_{SS})}{2C_{H}}]$$

Considering the substrate bias effect and $\gamma_{N}\approx \gamma_{P}=\gamma$. The output *V_o_* can be expressed as

(28) $$V_{o}\approx V_{in}(1+2K_{H}+\gamma K_{H}\frac{1}{\sqrt{V_{2}}})+K_{H}(V_{THN0}+V_{THP0})+\gamma K_{H}\frac{1}{16{\sqrt{V_{2}}}^{5}}V_{in}{}^{3}$$

The above Equation (28) shows that for CMOS complementary switches, the channel charge injection effect is still nonlinear and leads to harmonic generation. With the increase of switch size, the impact is intensified, so the switch size should be properly selected in the design. Obviously, the main reason why the channel charge injection effect brings nonlinearity to the system is the substrate bias effect. In order to effectively suppress the clock feedthrough effect and channel charge injection effect, we designed six-transistor CMOS complementary switch with virtual transistors as shown in Figure 6b. The transistor *M_1_* and *M_3_* constitute complementary switch, *M_2_* and *M_4_* as virtual transistors can absorb the channel injected charge when the clock is turned off. We can reasonably design the width-to-length ratio of virtual transistors to minimize the clock feedthrough effect. We optimally designed the parameters in switches and the first-stage integrator as shown in Table 2.

**Figure 7:**
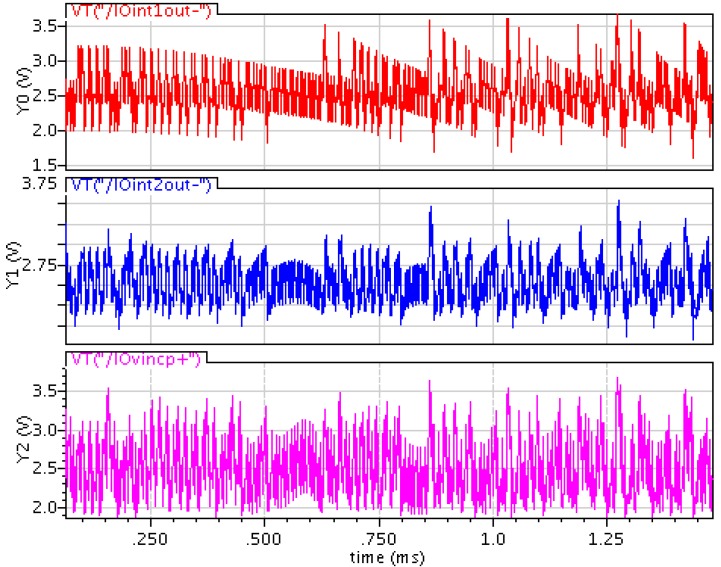
Outputs of the integrators.

After analyzing the harmonic distortion of interface circuit, the circuit parameters of each module are calculated and optimized. In order to verify the rationality of calculation and analysis, we use the high-speed parallel simulator in Cadence to verify the function of the whole system. We use 0.35 μm CMOS standard technology and set a simulated power supply voltage of 5 V. Because the typically output from TMR element is ac signal at the millivolt range. We set an input signal amplitude of 300 mV and a signal frequency of 250 Hz in simulation. We designed a closed-loop gain of 26 dB in the CFIA. The transient simulation output waveform of integrators at all levels is as shown in Figure 7. The waveforms in Figure 7 are the first level integrator, the second level integrator and the third level integrator from top to bottom respectively. It can be seen from the Figure 7 that the output of integrators at all levels is stable and the output swing is small.

**Figure 8:**
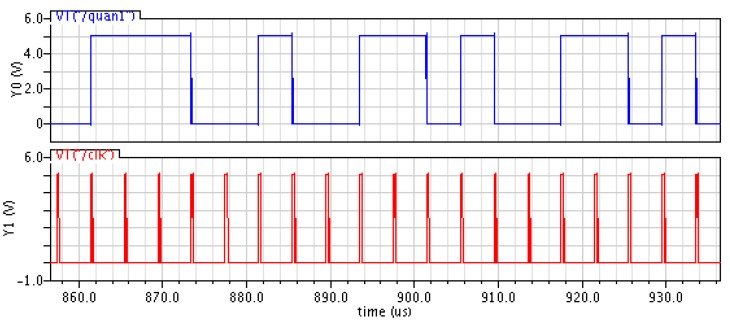
Outputs of the quantizer and sampling clock.

Figure 8 shows the output waveforms of sigma-delta quantizer and sampling clock respectively. When the rising edge of the sampling clock is valid, the quantizer starts to output. When the sampling clock is off, the output of the quantizer keeps the output of the previous time. It can be seen from the output waveform in the Figure 8 that using the sampling clock as a reference, the output of the quantizer does not have a continuous high or low level for a long time, which can show a good stability in the high-order sigma-delta system.

**Figure 9:**
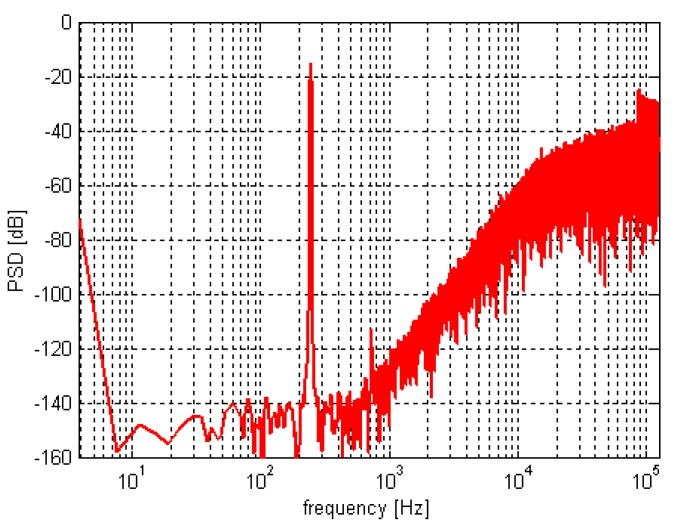
PSD of the quantizer output.

The sigma-delta TMR micro-sensors system (TMR sensitive element together with interface circuit) is simulated. We sample the output results of the quantizer at equal intervals and sample 65536 points for fast Fourier transform (FFT) analysis. The power spectral density (PSD) calculated and processed in MATLAB (R2016a, MathWorks, Natick, US) is shown in Figure 9. It can be seen from the results shown that the system realizes the function of noise shaping and the quantization noise at the low-frequency is shaped to the high-frequency. The noise floor level is lower than −140 dBV/Hz^1/2^. According to a reference voltage of ±2.5 V, the output noise voltage density in the signal band is lower than 250 nV/Hz^1/2^. Since the sensitivity of the sigma-delta TMR micro-sensors system is 0.1 V/Oe (1 Oe=10^−4^ T), the equivalent input noise of TMR sensors in the signal bandwidth is less than 0.25 nT/Hz^1/2^.

When the amplitude of input signal is large, the third harmonic distortion as shown in Figure 9 is less than −110 dB. In order to verify the performance of TMR sensors interface circuit, the ideal sensitive structure is used in the simulation. The interface circuit adopts the full differential structure, so it can be seen from output FFT results that the second harmonic distortion is not obvious in the circuit simulation. The third harmonic distortion mainly comes from the nonlinearity of the first-stage integrator and the switch.

## 3. Results

### 3.1. Interface Circuit Testing

**Figure 10:**
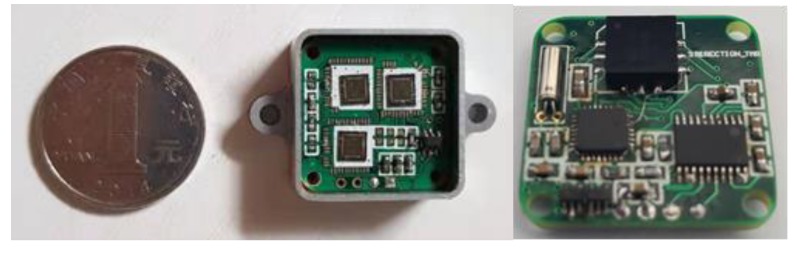
PCB photograph of three pathways interface circuit chip and TMR sensitive element.

**Figure 11:**
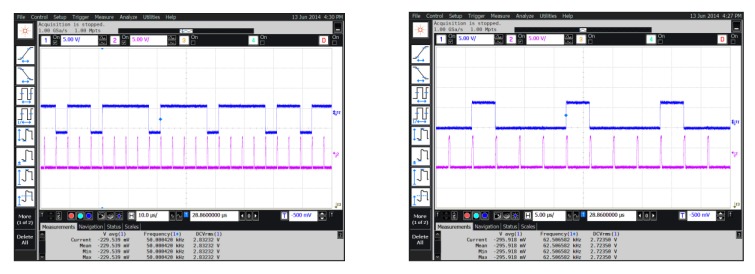
Transient response of sigma-delta modulator output.

**Figure 12:**
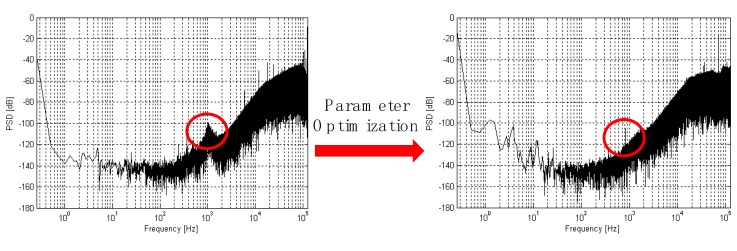
Comparison of test results before and after harmonic distortion optimized.

To verify the analysis presented in the previous sections, the interface circuit chip was designed in a standard 0.35 μm CMOS process and Figure 10 shows that the interface ASIC chip with three pathways (X-axis, Y-axis, and Z-axis) of the TMR sensors is made on the four-layer printed-circuit-board (PCB). The TMR sensitive element is on the opposite side of ASIC chip. The 46 pad pins for chip test on the interface ASIC chip are connected with the welding points on the corresponding PCB. The pad on the chip and the pad on the PCB are connected with silicon aluminum wire by the welding machine. The prototype was assembled on the non-magnetic aluminum box and then we tested the chip. The active area of the chip is 3.2 × 2 mm. We verified the function of the interface circuit before testing the performance of TMR sensors system. The digital bit stream output is collected from the Sigma-Delta interface circuit by the oscilloscope Agilent MSO9104A (Agilent Technologies Inc., Santa Clara, CA, USA). Transient response results of the interface circuit are shown in Figure 11. The results show that the interface circuit can achieve analog digital conversion function. We can verify the correctness of its function from the test results. The input signal and clock signal is supplied by Tektronix AFG3102 (Tek Technology Company, Shanghai, China) function signal generator. The 98000-point digital output sequence of the sigma-delta modulator is captured by an Agilent Logic analyzer 16804A (Agilent Technologies Inc., Santa Clara, CA, USA). The ouput digital signal is used to calculate the output power spectral density (PSD) as shown in Figure 12 by a MATLAB program. We optimized the switches and integrators in the sigma-delta modulator. Figure 12 shows the comparison of test results previous work with no optimization and after harmonic distortion optimized. The power dissipation of the interface circuit chip is 8.6mW at a sampling frequency of 6.4 MHz. The sigma-delta interface circuit has a dynamic range (DR) of 93 dB. The average noise floor in low-frequency range is less than -140 dB. The sigma-delta modulator can achieve an effective number of 18.6 bits.

We propose the third-order CIFF sigma-delta interface circuit which can get a better performance than most of the reported modulator in Table 3, compared with [19], although the FOM (Figure of Merit FOM=*P/BW×10^DR/20^*) of this work is smaller due to the disadvantage of process technology. This sigma-delta interface circuit satisfies the application in digital TMR sensors.

### 3.2. Test of TMR Sensors System

**Figure 13:**
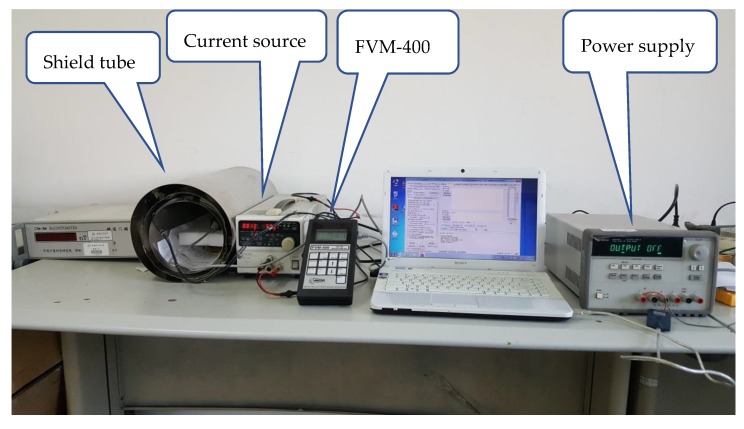
Test system of TMR sensors system.

**Figure 14:**
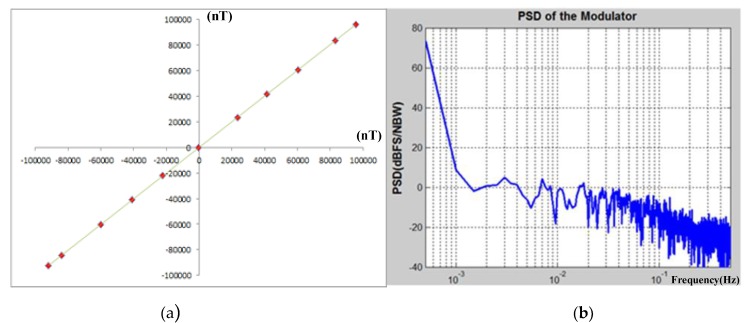
Test results of TMR micro-sensors: (**a**) linearity test of TMR micro-sensors system; (**b**) noise test of TMR micro-sensors system.

After testing the interface ASIC chip, we tested the TMR sensitive element together with the interface circuit. In order to avoid the disturbance of geomagnetic field and other electrical equipment magnetic field, we build the high-precision test platform as shown in Figure 13. The TMR magnetometers are put into a three-layer shielding tube made of high-magnetoconductivity permalloy. The magnetic field is adjustable by the constant-current source (Kenwood PW36-1.5ADP). The high-precision fluxgate magnetometer FVM-400 (MEDA Company High-resolution fluxgate, magnetic field resolution<0.1 nT(@1 Hz)) is useful for measuring the value of magnetic field. The power supply of the interface circuit combined with sensitive element is supported by the Agilent 3631A (Agilent Technologies Inc., Santa Clara, CA, USA). The test results are as shown in Figure 14. The full scale range is ±10^5^ nT, the TMR sensors system can achieve a linearity of 0.3% at full scale range as shown in Figure 14a. The Σ-Δ TMR micro-sensors system can achieve a power dissipation of 20 mW at a supply voltage of 5 V. Figure 14b shows the normalized noise test results which can achieve −11.22 dB at 1 Hz corresponding to 0.25 nT/Hz^1/2^(@1 Hz). The TMR sensors system can achieve a resolution of 0.25 nT/Hz^1/2^ over a signal bandwidth, which is limited by the low-frequency noise of the sensitive element. This work presents the parameters of TMR sensors system (TMR sensitive element together with interface circuit) as shown in Table 4. We propose this interface ASIC based on ΣΔ TMR micro-sensors can satisfy the high-precision application in digital micro-magnetometers.

## 4. Conclusions

In this work, we poposed a third-order fully differential sigma-delta interface circuit for tunneling magnetoresistance micro-sensors. In the sigma-delta circuit we optimized the CMOS switch and first-stage integrator in the switched-capacitor circuit. The interface circuit is fabricated in a standard 0.35 μm CMOS process. We tested the function and performance of interface circuit. The circuit can achieve a dynamic range (DR) of 93 dB and an average noise floor of less than -140 dB at low-frequency range. At last we tested TMR sensitive element with interface circuit, the results show that the TMR micro-sensors system can achieve a resolution of 0.25 nT/Hz^1/2^ and a linearity of 0.3% at ±10^5^ nT.

## Figures and Tables

**Table 1:** Parameters of TMR sensitive element.

Parameters	Value
Sensitivity	20 mV/V/Oe
Resistance	2 kΩ
Saturation magnetic field	±30 Oe
Hysteresis	0.02 Oe(@±2Oe)
Sensitivity temperature coefficient	-1100 ppm/℃
Resonance frequency	>1000 Hz
Noise floor	150 pT/Hz^1/2^

**Table 2:** Optimized parameters.

Switch size	Gain	Bandwidth	Swing Rate	*C_S_*	C*_f_*
12	68 dB	30 MHz	40 mV/s	4 pF	12 pF

**Table 3:** Performance summary and comparison

Parameters	[19]	[20]	[21]	[22]	This work
Bandwidth (Hz)	0.4 k	20 k	10 k	11 k	25 k
Peak SNDR (dB)	104.9	88.7	-	62	81
DR (dB)	-	99	70.2	80	93
Supply (V)	5	3.3	0.9	1.8	5
Power (mW)	50	5.6	0.06	1.7	8.6
Process (µm)	0.6	0.18	0.18	0.5	0.35
FOM	-	3.14	1.85	15.4	7.7

**Table 4:** Performance of TMR sensors system

Properties	Values
Supply voltage	5 V
Process technology	0.35 μm CMOS
Measuring range	±100 μT
Nonlinearity	0.3%
Power consumption	20 mW
Chip area	6.4 mm^2^
Resolution	<1 nT(@1Hz)
